# Heart rate variability changes associated with wetsuit hood fit in dry conditions: a pilot exploratory study

**DOI:** 10.3389/fphys.2026.1759395

**Published:** 2026-07-08

**Authors:** Enrico Moccia, Laura Stefani, Fabio di Pumpo, Fabio Gobbi, Gualtiero Meloni, Luigi Formichini, Lorenzo Rondinini, Vincenzo Rizzuto, Francesco Schiavone

**Affiliations:** 1 Medical Officer of the Divers and Special Forces Command of the Italian Navy, Le Grazie, Portovenere, La Spezia (SP), Italy; 2 Sports Medicine Center-University of Florence, Azienda Ospedaliero Universitaria Careggi (AOUC), Florence, Italy; 3 Retired, Florence, Italy; 4 Inspectorate of the Italian Navy, Rome, Italy; 5 School of Advanced Studies, Center for Neuroscience, University of Camerino, Camerino, Italy; 6 Department of Ophthalmology, Riga Stradins University, Riga, Latvia; 7 Department of Biomedical Sciences and Public Health, Marche Polytechnic University, Ancona, Italy

**Keywords:** diving physiology, heart rate variability, HRV, kubios HRV, pNN50, RMSSD, SDNN, wetsuit hood

## Abstract

**Background:**

This pilot study explored whether wearing wetsuit hoods is associated with short-term changes in heart rate variability (HRV)-derived indices under controlled dry laboratory conditions.

**Methods:**

Eleven healthy male military participants underwent HRV recordings across four fixed-sequence conditions (Baseline, White, Blue, Yellow), administered in an order corresponding to different nominal hood sizes and expected levels of fit. RR recordings were acquired using the Polar H10 sensor and processed in *Kubios HRV Premium* with standardized artifact correction (medium filter), yielding normal-to-normal (NN) intervals for analysis. HRV measures included SDNN, RMSSD, pNN50, and *Kubios*-derived PNS/SNS composite indices. Mechanical compression was not directly measured; exploratory analyses considered hood fit using geometric mismatch proxies.

**Results:**

Several HRV-derived indices differed significantly across conditions (SDNN p = 0.036, RMSSD p = 0.011, pNN50 p = 0.004; PNS p = 0.034; SNS p = 0.049). The most consistent pattern involved reductions in RMSSD and pNN50 under hooded conditions, together with changes in composite *Kubios*-derived indices. Relative metrics (%PNS/%SNS) remained unchanged. Exploratory analyses suggested possible associations between hood fit and selected HRV-derived measures; however, continuous-strain analyses did not identify a robust dose–response relationship, and within-subject variation was confounded by experimental sequence order.

**Conclusions:**

Under controlled dry laboratory conditions, wearing wetsuit hoods was associated with differences in selected HRV-derived indices. These findings should be interpreted as exploratory and hypothesis-generating rather than mechanistic, as the present design does not permit attribution to specific physiological pathways. Future studies incorporating randomized designs, direct pressure measurements, and respiratory monitoring are required to clarify the reproducibility and physiological basis of hood-associated HRV changes.

## 1 Introduction

The autonomic nervous system contributes to physiological adaptation during diving through complex cardiovascular and respiratory responses, including the diving reflex and associated vagally mediated mechanisms triggered by facial immersion (Schaller, 2004; Panneton, 2013; Lemaitre et al., 2015). Heart rate variability (HRV) has been widely used to characterize cardiovascular variability during diving, immersion, and hyperbaric exposure (Schipke et al., 2001; Christoforidi et al., 2012; Noh et al., 2018; Peláez-Coca et al., 2024). However, contemporary methodological literature emphasizes that HRV-derived indices should not be interpreted as direct measures of sympathetic or parasympathetic activity, and attributing observed changes to specific physiological mechanisms remains challenging (Berntson et al., 1997; Eckberg, 1997; Goldberger, 1999; Houle and Billman, 1999; Malpas, 2002; Goldstein et al., 2011; Rahman et al., 2011; Billman, 2013a; Reyes del Paso et al., 2013; Heathers, 2014; Billman et al., 2015; Quintana et al., 2016; Laborde et al., 2017; Quigley et al., 2024). Previous studies have reported HRV alterations during immersion, breath-hold diving, and SCUBA exposure (Schipke et al., 2001; Christoforidi et al., 2012; Noh et al., 2018; Lundell et al., 2020; Lundell et al., 2021; Ackermann et al., 2023; Lundell and Ojanen, 2023), although these responses are influenced by multiple interacting factors including respiration, thermal stimuli, hydrostatic pressure, and psychological adaptation (Berntson et al., 1997; Grossman and Taylor, 2007; Shattock and Tipton, 2012; Quintana and Heathers, 2014; Quigley et al., 2024). Despite extensive investigation of HRV in diving environments, no previous study has specifically examined whether cranial–cervical constraint associated with wetsuit hood fit is related to short-term changes in HRV under controlled dry conditions. The Mephisto hood, commonly used in military and recreational diving, may impose different degrees of cranial and cervical constraint depending on individual fit; however, potential mechanisms underlying associated physiological responses remain speculative and were not directly assessed in the present study. Therefore, this pilot study explored whether wearing differently fitting wetsuit hoods is associated with short-term changes in selected HRV-derived indices under standardized dry laboratory conditions. Given current methodological concerns regarding mechanistic interpretation of HRV (Berntson et al., 1997; Eckberg, 1997; Goldberger, 1999; Houle and Billman, 1999; Malpas, 2002; Goldstein et al., 2011; Rahman et al., 2011; Billman, 2013a; Reyes del Paso et al., 2013; Heathers, 2014; Quintana et al., 2016; Laborde et al., 2017; Quigley et al., 2024), the study was designed as exploratory and hypothesis-generating rather than as a test of specific autonomic mechanisms. We hypothesized that hood fit may be associated with changes in selected HRV-derived indices, potentially influenced by individual anatomical characteristics and mechanical constraint.

## 2 Materials and methods

### 2.1 Participants

Eleven healthy, physically active adult volunteers (active military personnel) were enrolled. We recorded sex, age, stature, body mass (to compute BMI) (**Supplementary Table 2**), and neck circumference to account for potential differences in hood fit and mechanical constraint. Inclusion criteria were: age 18–65 years, current fitness-to-dive clearance, absence of cardiovascular, neurological, or respiratory disease and no use of cardioactive medication. Exclusion criteria included acute illness in the prior 2 weeks, history of syncope, or arrhythmia. Participants were instructed to refrain from caffeine, alcohol and strenuous exercise for 24 h before testing.

### 2.2 Design and protocol

**Figure 1:**
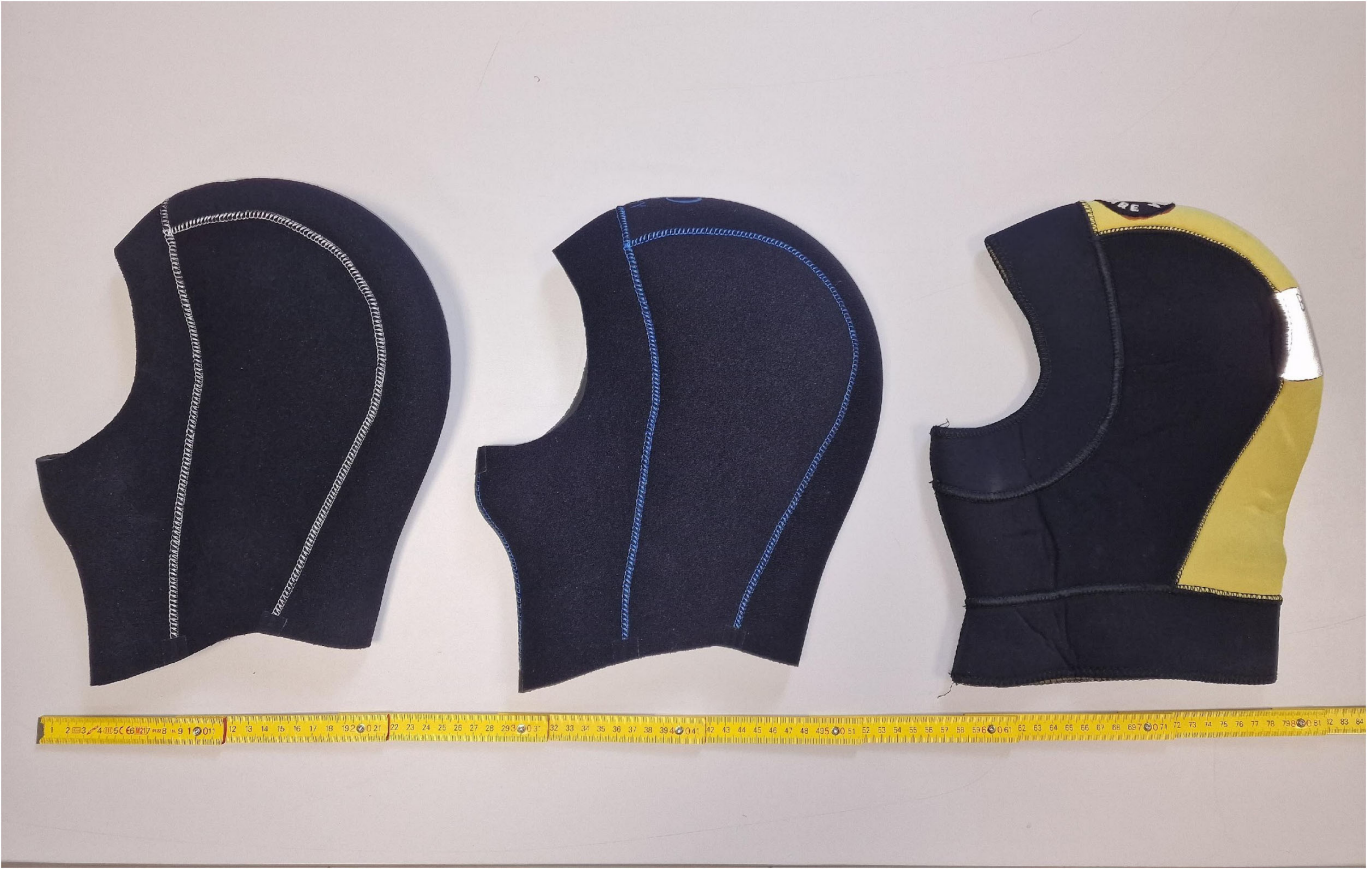
Three Mephisto hood configurations used in the study (White, Blue, Yellow), corresponding to different nominal hood sizes and expected differences in hood fit.

This was a fixed-sequence, within-subject crossover study with four experimental conditions (Baseline; Mephisto White; Blue; Yellow), administered in an order corresponding to different nominal hood sizes and expected levels of cranial–cervical fit. Each recording was performed under dry conditions, with participants seated in an air-conditioned room. The first phase was conducted without a hood to establish the individual baseline HRV reference value, followed by three phases in which participants wore Mephisto hoods of different sizes, corresponding to different nominal hood sizes and expected levels of fit (**Figure 1**). Each phase lasted 8 minutes, a duration selected to allow reliable analysis of linear and non-linear HRV parameters in *Kubios Premium*. To reduce time-related drifts, we inserted 5-min seated washouts between phases, scheduled all recordings at a consistent time-of-day and instructed participants to abstain from caffeine, alcohol and strenuous exercise for 24 h before testing. The sequence of conditions was fixed (Baseline → White → Blue → Yellow) to standardize hood exposure across participants. While this approach allowed within-subject comparability, it did not control for potential order effects, which are therefore acknowledged as a study limitation. The primary outcome of the study was the within-subject change in RMSSD (root mean square of successive NN interval differences) across conditions. This parameter was selected because it is commonly interpreted as a marker of short-term vagally mediated HRV and is widely used in psychophysiological research (Billman, 2013b; Laborde et al., 2017). However, RMSSD is influenced by respiratory rate through respiratory sinus arrhythmia. In the present study, respiratory frequency was neither measured nor controlled, which may have affected the interpretation of vagally mediated HRV changes across conditions. In addition to RMSSD, we evaluated SDNN (standard deviation of NN intervals), which reflects overall heart rate variability, and pNN50 (percentage of adjacent NN intervals differing by more than 50 ms), another commonly used index of short-term vagally mediated HRV. Finally, to complement the time-domain measures, we analyzed the *Kubios*-derived sympathetic (SNS) and parasympathetic (PNS) indices, which represent composite HRV-derived metrics generated by Kubios software from multiple physiological features. This study was conceived as a pilot within-subject crossover primarily aimed at feasibility assessment and preliminary estimation of variability and effect sizes. Consequently, no formal *a priori* sample-size calculation was performed.

This study was conducted in accordance with the principles of the Declaration of Helsinki. The protocol was reviewed and approved by the Pisa University Bioethics Committee (Resolution n. 69/2024, 17 December 2024). All participants were active military personnel who volunteered and provided written informed consent before participation.

### 2.3 Equipment and data acquisition

R–R intervals were recorded using a Polar H10 chest strap (Polar Electro Oy, Kempele, Finland) with a sampling frequency of 1,000 Hz, which provides accuracy comparable to standard electrocardiography under resting conditions. Data were exported and analyzed using *Kubios HRV Premium* software (Kubios Oy, Kuopio, Finland). RR intervals were preprocessed with automatic artifact correction set to “Medium”. Ectopic beats were corrected by cubic spline interpolation, resulting in normal-to-normal (NN) intervals used for HRV analysis.

### 2.4 Wetsuit hoods

Three hand-made hoods of identical construction and different nominal sizes (M, L, XL) were used (PHY Hood, PHY Diving Equipment, Italy; nominal thickness 5 mm). The material consisted of Japanese limestone-based closed-cell foamed polychloroprene characterized by a 93–94% closed-cell nitrogen-filled microstructure and lower bulk density compared with petroleum-based neoprene. Material properties were not measured directly on tested hoods but derived from published data on equivalent commercial neoprene, including bulk density (≈195–235 kg·m^-^³), mass per unit area (≈1100–1300 g·m^-^² at 5 mm), and elongation at break (≈480–580%) (43). For study purposes, the three hoods were identified by color code (White, Blue, Yellow), corresponding to different nominal sizes and expected differences in hood fit. As direct pressure measurements were not performed, compression was operationally treated as a relative subject-specific variable. Internal hood dimensions were assessed by measuring head-level and neck-level circumferences directly on each hood under standardized conditions. Individual anthropometric parameters (maximum head circumference and neck circumference) were measured for each participant using the same protocol. All measurements were performed in triplicate and averaged to improve reliability. Mechanical constraint was therefore explored using subject-specific geometric indices derived from mismatch between anthropometric measurements and hood internal dimensions (see Section 2.3). These indices do not account for material elasticity, tissue compliance or dynamic deformation during wear and therefore represent approximations of geometric mismatch rather than direct biomechanical measurements. Results based on these parameters should consequently be interpreted with caution.

#### 2.4.1 HRV measurement

HRV analysis included SDNN (standard deviation of NN intervals), RMSSD (root mean square of successive NN interval differences), and pNN50 (percentage of adjacent NN intervals differing by >50 ms). RMSSD and pNN50 are commonly interpreted as indices of short-term vagally mediated HRV, whereas SDNN reflects overall variability in NN intervals and should not be considered a specific marker of sympathetic activity (Berntson et al., 1997; Eckberg, 1997; Houle and Billman, 1999; Malpas, 2002; Goldstein et al., 2011; Rahman et al., 2011; Billman, 2013a; Reyes del Paso et al., 2013; Quigley et al., 2024). In accordance with contemporary methodological recommendations, frequency-domain measures (LF, HF, LF/HF) were not interpreted as direct indicators of sympathovagal balance (Eckberg, 1997; Goldberger, 1999; Billman, 2013a; Heathers, 2014; Quigley et al., 2024). Because these variables were not pre-specified primary outcomes, they were excluded from inferential analyses and logarithmic transformation was not performed. This approach was adopted to limit multiple testing in the context of an exploratory pilot study. Interpretation of HRV findings additionally considered prevailing heart rate, which may mathematically influence HRV indices independently of autonomic processes (Billman et al., 2015). Accordingly, descriptive heart rate measures (Mean HR, Mean RR, HRmin, and HRmax) were examined to contextualize HRV changes. The Kubios-derived SNS and PNS indices are proprietary composite metrics integrating multiple HRV features, including mean heart rate, RMSSD, SD1, and Baevsky stress index. These measures were interpreted as indirect descriptors of HRV patterns rather than direct indicators of sympathetic or parasympathetic activation. Alternative threshold-based HRV metrics (e.g., pNNx measures beyond pNN50) and very-low-frequency (VLF) components were not explored. Although interpretation of these measures in short-term recordings remains debated, they may provide complementary information in future investigations (Mietus et al., 2002).

#### 2.4.2 Statistical analysis

**Figure 2:**
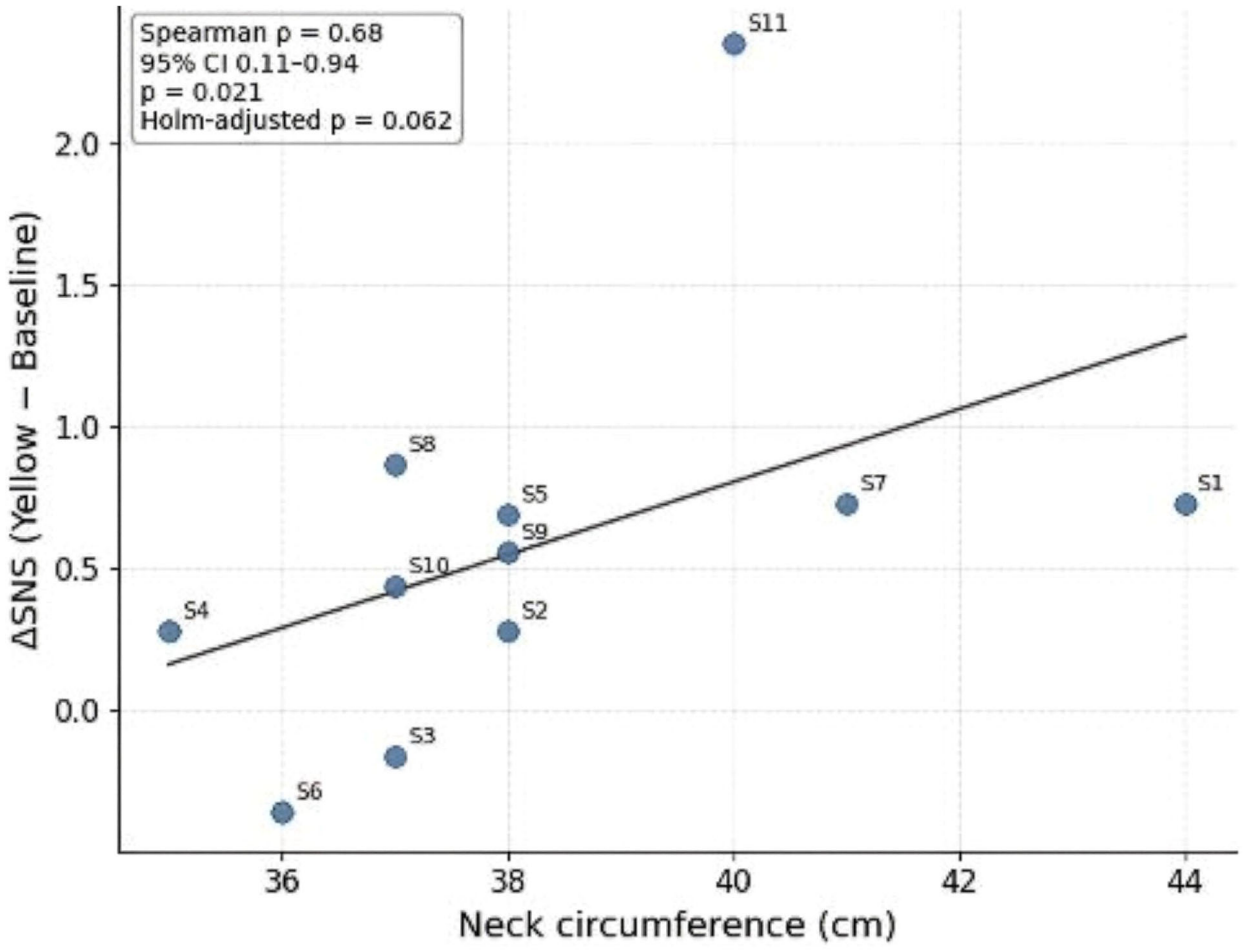
Association between neck circumference and changes in the Kubios-derived SNS metric under the Yellow hood condition. Scatter plot showing ΔSNS (Yellow − Baseline) versus neck circumference (cm). Each point represents one participant. The solid line indicates the least-squares fit and is shown for visualization purposes only; associations were assessed using Spearman’s rank correlation. Larger neck circumference was associated with greater changes in the Kubios-derived SNS metric under the Yellow condition (Spearman’s ρ = 0.68; 95% CI 0.11–0.94; unadjusted p = 0.021; Holm-adjusted p = 0.062). Given the exploratory nature of the analysis and the small sample size, results should be interpreted cautiously.


To assess differences among the four experimental conditions (Baseline, White, Blue, Yellow), normality was first evaluated using the Shapiro–Wilk test. Because some variables were not normally distributed, Friedman tests (**Table 1**) were used for repeated-measures comparisons. When omnibus tests were significant, *post-hoc* pairwise comparisons were performed using Wilcoxon signed-rank tests (**Supplementary Table 1**). P-values from pairwise tests were adjusted using Holm correction for multiple comparisons. To quantify effect magnitude, Kendall’s W was reported for Friedman tests and standardized effect size r for Wilcoxon comparisons, computed as r = Z/√N (with Z obtained from the two-sided normal approximation and N equal to the number of non-zero pairs). Two-tailed significance was set at p < 0.05. To explore whether interindividual fit contributed to changes in HRV-derived indices, associations between neck circumference (NC) and within-subject changes in HRV indices (Δ = condition − Baseline) for White, Blue and Yellow hoods were examined (**Figure 2**). Spearman rank correlations (ρ) were computed together with 95% bootstrap confidence intervals (2,000 resamples). P-values were adjusted using Holm correction within each HRV family. As a complementary exploratory approach leveraging repeated measures, linear mixed-effects models of the form:

**Table 1:** Results of the Friedman test for repeated-measures analysis across the four experimental conditions (Baseline, White, Blue, Yellow).

Parameters	Statistics	p-value
SDNN	8.56	0.036*
RMSSD	11.20	0.011*
PNN50	13.53	0.004*
%PNS	2.38	0.497
%SNS	2.38	0.497
PNS	7.56	0.034*
SNS	6.94	0.049*

Statistically significant differences (p < 0.05) were observed for SDNN, RMSSD, pNN50, PNS, and SNS, indicating variations in selected HRV-derived indices across conditions. No significant differences were found for %PNS and %SNS. (*significant value).

Y~condition×NC+(1|id)

were fitted for RMSSD and the Kubios-derived SNS metric. Analyses were performed in MATLAB R2023b (The MathWorks, Natick, MA, USA).

#### 2.4.3 Compression characterization and strain–HRV analysis

Because nominal hood size did not consistently correspond to subject-specific cranial–cervical constraint, categorical-condition analyses were complemented by continuous-strain analyses. For each subject–hood combination, a head strain index was computed as:

Strain_head (%) = (HC_subj − HC_hood)/HC_hood ×100

where HC_subj is participant head circumference and HC_hood the internal hood circumference measured at forehead level. Neck strain was computed analogously from NC_subj and NC_hood.

Positive values indicate relative compression, whereas values ≤0 indicate loose fitting without apparent mechanical compression. Across 33 subject–hood combinations, head strain ranged from 21.2% to 54.2%, with consistent ordering across conditions (Yellow > Blue > White). Neck strain ranged from −13.8% to +30.9%; 17/33 observations had strain_neck ≤0. Because the experimental sequence proceeded from widest to narrowest hood (Baseline → White → Blue → Yellow), within-subject strain variation was collinear with sequence order (**Supplementary Figure 1**).

Linear mixed-effects models:


ΔHRV∼strain+(1|subject)

were fitted for ΔSDNN, ΔRMSSD, ΔpNN50, ΔSNS, and ΔPNS using REML estimation in MATLAB. Results are reported as β coefficients, standard errors, 95% confidence intervals, p-values, and marginal/conditional R². To separate between-subject from within-subject strain effects, strain was decomposed into subject-level mean strain and within-subject deviation:


ΔHRV∼strain_between+strain_within+(1|subject)

A diagnostic analysis was additionally performed on observations with strain_neck ≤0 (absence of apparent neck compression). In this subset, observed changes from baseline are unlikely to be explained solely by apparent cervical compression. One-sample Student’s t-tests and Wilcoxon signed-rank tests were performed against a null hypothesis of zero change.

## 3 Results

**Figure 3:**
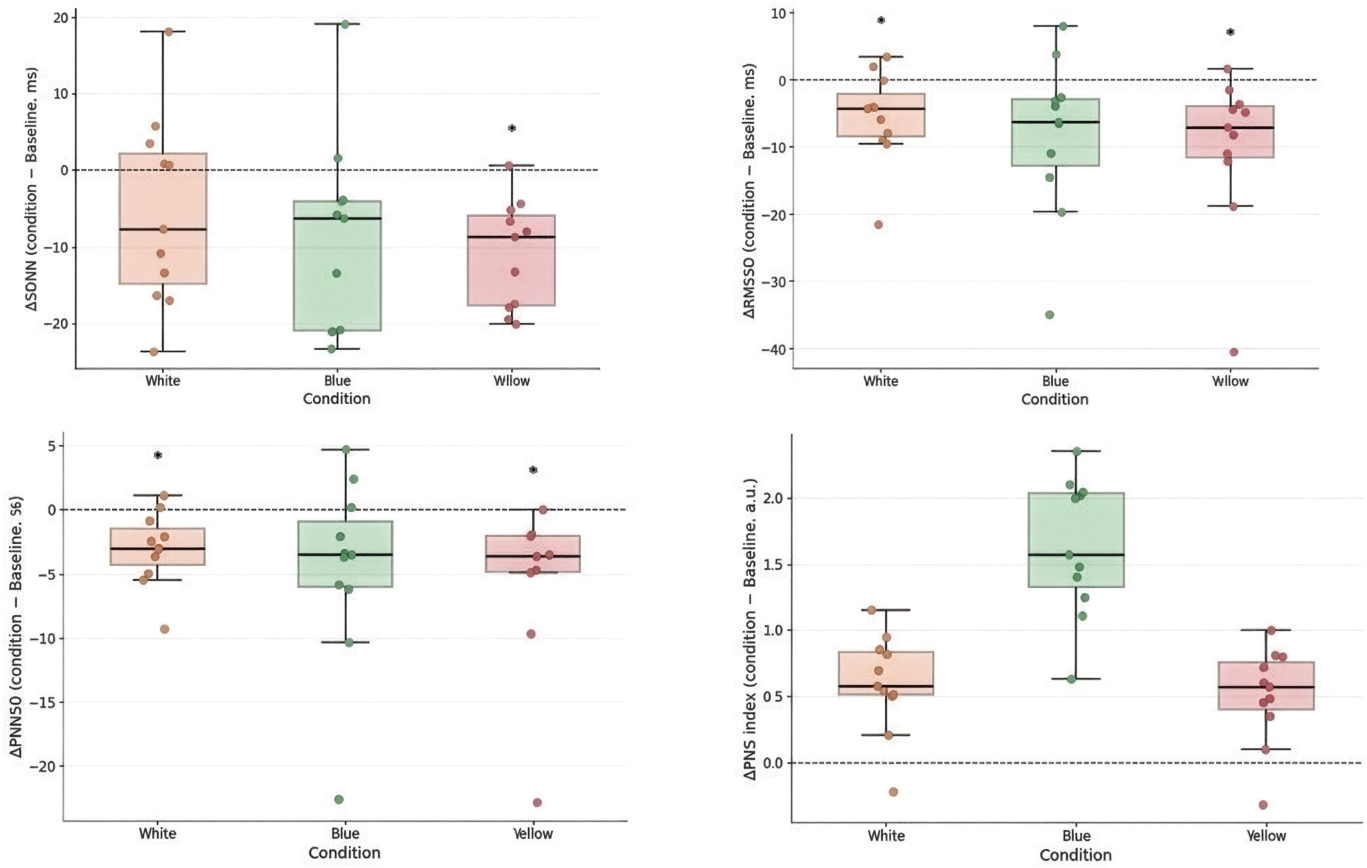
Change-from-baseline (Δ) HRV-derived indices across hood conditions (White, Blue, Yellow). For each participant, Δ values were calculated as condition − Baseline. Panels show ΔSDNN (top left), ΔRMSSD (top right), ΔpNN50 (bottom left), and ΔPNS index (bottom right). The horizontal dashed line at 0 indicates no change from Baseline. Boxplots represent median and interquartile range, with individual data points overlaid. Significant reductions after Holm correction were observed for RMSSD in the White and Yellow conditions and for pNN50 in the White and Yellow conditions, whereas Blue-condition differences did not remain significant after correction. SDNN showed a significant reduction only in the Yellow condition. No significant differences were observed for the Kubios-derived PNS index after multiple comparison correction. Overall, these findings indicate reductions in selected HRV-derived measures under some hood conditions relative to Baseline, although interpretation should remain cautious given the exploratory design and limited sample size. * Indicates conditions that remained significantly different from Baseline following Holm-adjusted post-hoc Wilcoxon signed-rank tests (adjusted p < 0.05).

Eleven male participants, all active members of the Italian Navy with non-competitive athletic backgrounds, completed the study. Mean age was 44.5 ± 9.1 years (range 30–61), mean height 1.76 ± 0.08 m (range 1.62–1.85), mean body mass 84.5 ± 12.1 kg (range 66–105), and mean BMI 27.1 ± 3.5 kg/m² (range 22.84–33.91). Neck circumference averaged 38.3 ± 2.5 cm (range 35–44). Descriptive participant characteristics are reported in **Supplementary Table 2**. All HRV recordings were successfully acquired and no recordings were excluded after artifact correction using the Kubios Premium “Medium” filter. Descriptive heart rate measures (Mean HR, Mean RR, HRmin, HRmax) are reported in **Supplementary Table 3**. Mean HR differed across experimental conditions at the omnibus level (Friedman χ² = 8.13, p = 0.043), although pairwise comparisons versus Baseline were not significant after correction. Significant differences across conditions were observed for SDNN (p = 0.036), RMSSD (p = 0.011), pNN50 (p = 0.004), PNS (p = 0.034), and SNS (p = 0.049) (**Table 1**), whereas %PNS and %SNS did not differ significantly (p = 0.497). *Post-hoc* Wilcoxon signed-rank tests (**Supplementary Table 1**) showed reductions in SDNN in the Blue (p = 0.024) and Yellow (p = 0.002) conditions relative to Baseline (**Figure 3**). RMSSD decreased in the White (p = 0.010), Blue (p = 0.042), and Yellow (p = 0.003) conditions. Similarly, pNN50 was reduced between Baseline and White (p = 0.007) and between Baseline and Yellow (p = 0.005), with an additional difference between White and Yellow (p = 0.011). PNS values did not differ significantly after correction, although the Baseline–Yellow comparison showed a trend toward lower values (p = 0.085). Conversely, SNS values were higher in the White (p = 0.032) and Yellow (p = 0.009) conditions relative to Baseline (**Figure 3**). The most consistent pattern involved reductions in RMSSD and pNN50 across hooded conditions together with changes in Kubios-derived indices, whereas %PNS and %SNS remained unchanged. Friedman analyses indicated small-to-moderate effects for SDNN (W = 0.26), RMSSD (W = 0.34), pNN50 (W = 0.41), PNS (W = 0.26), and SNS (W ≈ 0.24). Percent-based indices (%PNS, %SNS) showed negligible effects (W ≈ 0.07). Detailed effect sizes and Holm-adjusted p-values are reported in **Table 2**.

**Table 2:** Pairwise comparisons versus Baseline for key HRV indices (N=11).

Variable	Comparison	N_pairs	Wilcoxon_T	p_value	p_Holm	r_effect
RMSSD	Baseline vs White	11	5.0	0.00977	0.0195	-0.779
RMSSD	Baseline vs Blue	11	10.0	0.042	0.042	-0.613
RMSSD	Baseline vs Yellow	11	2.0	0.00293	0.00879	-0.897
PNN50	Baseline vs White	11	4.0	0.00684	0.0137	-0.816
PNN50	Baseline vs Blue	11	11.0	0.0537	0.0537	-0.582
PNN50	Baseline vs Yellow	10	0.0	0.00195	0.00586	-0.979
SDNN	Baseline vs White	11	20.0	0.278	0.278	-0.327
SDNN	Baseline vs Blue	11	8.0	0.0244	0.0488	-0.679
SDNN	Baseline vs Yellow	11	1.0	0.00195	0.00586	-0.934
SNS	Baseline vs White	11	9.0	0.0322	0.0645	0.646
SNS	Baseline vs Blue	9	9.0	0.129	0.129	0.506
SNS	Baseline vs Yellow	11	5.0	0.00977	0.0293	0.779

Wilcoxon signed-rank tests with Holm-adjusted p-values and standardized effect size r (r = Z/√N). Negative r indicates a decrease from Baseline; positive r indicates an increase. Abbreviations: SDNN, standard deviation of NN intervals; RMSSD, root mean square of successive differences; pNN50, percentage of adjacent NN intervals differing by >50 ms; SNS, sympathetic index derived in Kubios. Holm adjustment applied within each variable across the three planned contrasts.

### 3.1 Association with neck circumference

Neck circumference showed a positive association with changes in the Kubios-derived SNS metric in the Yellow condition (the last hood exposure after Baseline). Specifically, ΔSNS in the Yellow condition was positively correlated with NC (Spearman ρ = 0.68; 95% CI 0.11–0.94; unadjusted p = 0.021), although this association did not remain statistically significant after Holm correction (p = 0.062). This suggests a trend toward greater changes in the Kubios-derived SNS metric among participants with larger neck circumference during the final hood exposure (**Figure 2**). A more comprehensive analysis of the relationship between compression and ΔHRV across all HRV indices, using a continuous strain index that jointly accounts for head and neck dimensions, is reported in Section 3.2.

### 3.2 Compression–HRV analysis (continuous strain predictor)

**Figure 4:**
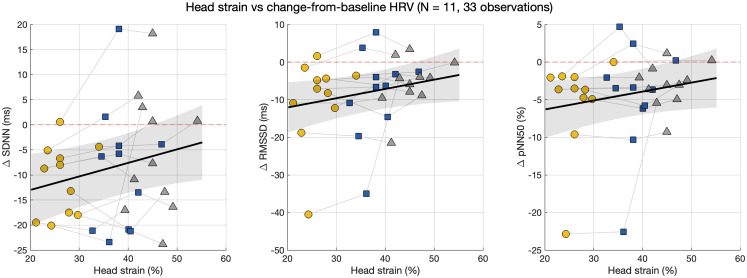
Head strain vs change-from-baseline HRV (N = 11, 33 observations). Scatter plots of head strain (%) versus within-subject change from baseline for SDNN, RMSSD and pNN50. Each point represents a single subject–condition observation, coloured and shaped by condition (yellow circles = White hood, first in temporal sequence; blue squares = Blue, second; grey triangles = Yellow, third). Thin grey lines connect the three observations of each subject in order of increasing strain. The solid black line and shaded band represent the fixed-effect regression line and 95% confidence interval from the linear mixed-effects model ΔHRV ~ strain + (1|subject). Inset: fixed-effect coefficient β, standard error, p-value (Satterthwaite dof) and marginal R².

**Figure 5:**
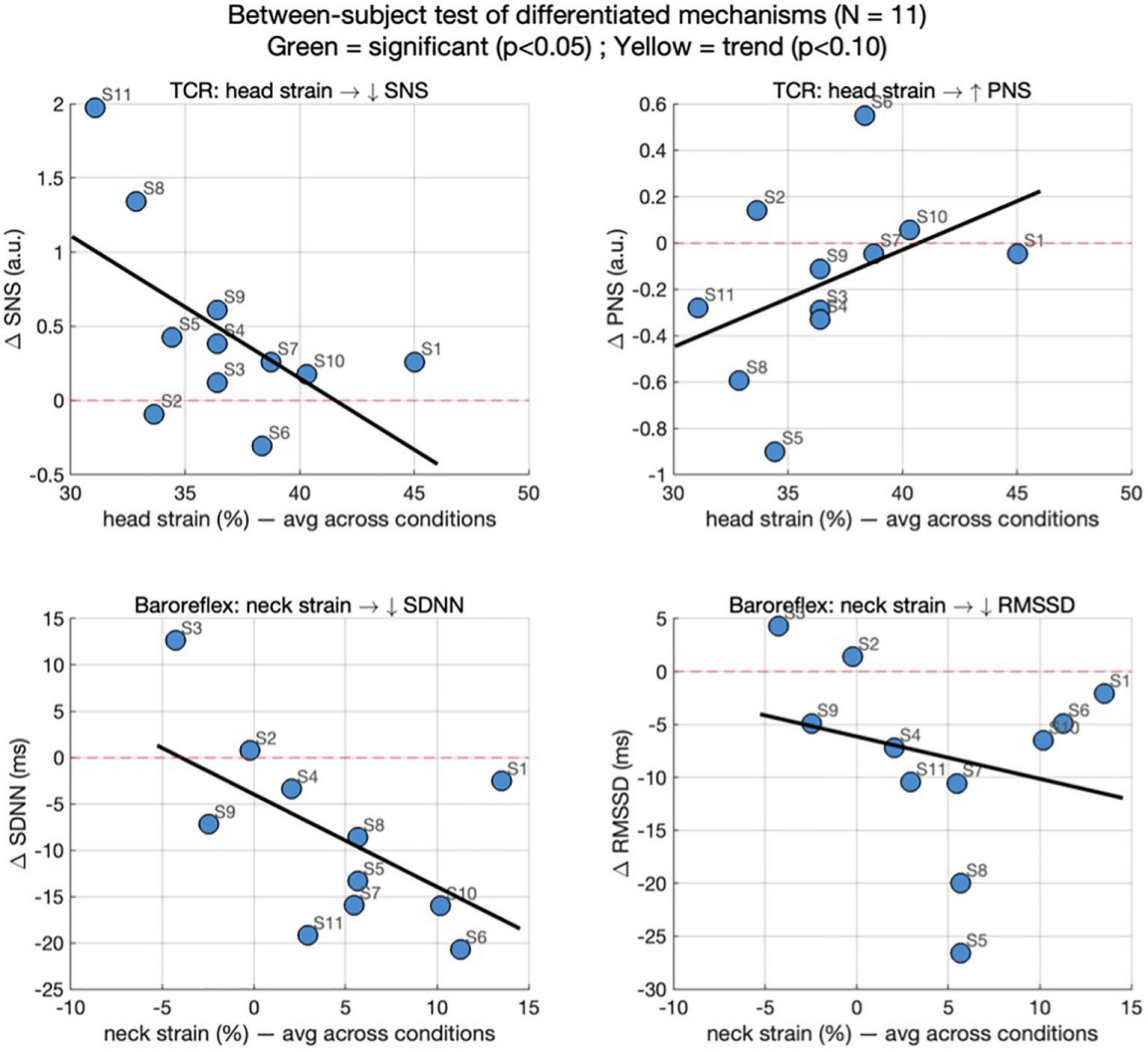
Between-subject associations between strain and change-from-baseline HRV (N = 11). Each point corresponds to one participant, plotted at their subject-level mean strain (head in the top row, neck in the bottom row) and mean ΔHRV (averaged across the three hood conditions). Top-left: ΔSNS vs head strain; top-right: ΔPNS vs head strain; bottom-left: ΔSDNN vs neck strain; bottom-right: ΔRMSSD vs neck strain. Solid line: least-squares linear fit. Dashed red line: zero change from baseline. Annotation boxes report the slope β, Pearson correlation r, and p-value; green background indicates p < 0.05, yellow indicates a trend at p < 0.10.

Across the 33 subject–condition observations, linear mixed-effects models of ΔHRV on head strain showed a significant positive slope for ΔSDNN (β = +0.27 ms per 1% strain, SE 0.13, 95% CI [+0.01, +0.53], p = 0.041, R²marginal = 0.04, R²conditional = 0.74), with trend-level positive slopes for ΔRMSSD (β = +0.25, p = 0.074) and ΔpNN50 (β = +0.12, p = 0.101). Composite Kubios indices showed small and non-significant slopes (ΔSNS: β = −0.009, p = 0.280; ΔPNS: β = +0.004, p = 0.517). Analogous models with neck strain as predictor yielded weaker associations (ΔSDNN: β = +0.16, p = 0.064; all other indices p > 0.10) (**Table 3**, **Figure 4**). Because the experimental sequence proceeded from the widest to the narrowest hood (White → Blue → Yellow), within-subject strain variation was collinear with sequence order; a positive slope in this pooled model is therefore compatible both with progressive habituation and with alternative compression-related mechanisms. To disambiguate between-subject (anatomical, not confounded with order) and within-subject (confounded with order) contributions, strain was decomposed into these two components and entered simultaneously as predictors (**Table 4**, **Figure 5**). Within-subject effects recapitulated the pooled-model findings, with the strongest effect for ΔSDNN (β_within = +0.27, p = 0.041). Between-subject effects showed a site-specific pattern: head strain was significantly associated with ΔSNS (β_between = −0.096, p = 0.046), with subjects of larger average head strain showing smaller increases in the Kubios-derived SNS metric; neck strain was significantly associated with ΔSDNN (β_between = −1.00, p = 0.049), with subjects of larger average neck strain showing greater reductions in global variability. All seven between-subject contrasts with a specific directional prediction aligned with the predicted sign (binomial sign test p < 0.01), although only two reached statistical significance, consistent with the limited power of between-subject analyses at N = 11. In the diagnostic subset of 17 observations in which the hood did not mechanically compress the neck (strain_neck ≤ 0; 8 in Blue, 9 in Yellow), significant changes from Baseline were nevertheless observed for all HRV-derived indices (**Table 5**): ΔSDNN = −9.4 ms (p = 0.003), ΔRMSSD = −10.7 ms (p = 0.003), ΔpNN50 = −5.6% (p = 0.007), ΔSNS = +0.61 (p = 0.001), and ΔPNS = −0.30 (p = 0.011). The persistence of significant changes in HRV-derived indices despite the absence of cervical compression suggests that direct cervical mechanical engagement alone is unlikely to explain the observed pattern.

**Table 3:** Mixed-effects models: ΔHRV vs head and neck strain.

HRV index	Predictor	β	SE	95% CI	p	R²marg	R²cond
ΔSDNN	Head	+0.270	0.126	[+0.012, +0.528]	0.041 *	0.04	0.74
ΔRMSSD	Head	+0.247	0.133	[−0.025, +0.518]	0.074 †	0.05	0.64
ΔpNN50	Head	+0.119	0.070	[−0.025, +0.262]	0.101	0.03	0.70
ΔSNS	Head	−0.009	0.008	[−0.025, +0.008]	0.280	0.01	0.73
ΔPNS	Head	+0.004	0.006	[−0.008, +0.015]	0.517	0.01	0.65
ΔSDNN	Neck	+0.165	0.086	[−0.010, +0.340]	0.064 †	0.03	0.76
ΔRMSSD	Neck	+0.143	0.090	[−0.042, +0.327]	0.125	0.03	0.67
ΔpNN50	Neck	+0.078	0.048	[−0.019, +0.174]	0.112	0.03	0.71
ΔSNS	Neck	−0.0003	0.006	[−0.012, +0.011]	0.950	0.00	0.73
ΔPNS	Neck	+0.002	0.004	[−0.006, +0.010]	0.641	0.00	0.65

Mixed-effects models ΔHRV ~ strain + (1|subject), fitted separately for each HRV index and each predictor (head strain, neck strain). β is the fixed-effect slope (HRV units per 1% strain), SE its standard error. 95% confidence interval from fixed-effect covariance. p-values from Satterthwaite-approximated degrees of freedom. R²marg = marginal (fixed effects only); R²cond = conditional (fixed + random). Significance: *p < 0.05; †p < 0.10.

**Table 4:** Between-subject / within-subject decomposition of strain effects on ΔHRV.

HRV index	Predictor site	β between	p between	β within	p within
ΔSDNN	Head	+0.150	0.862	+0.273	0.041 *
ΔRMSSD	Head	+0.815	0.266	+0.226	0.107
ΔpNN50	Head	+0.240	0.585	+0.115	0.117
ΔSNS	Head	−0.096	0.046 *	−0.007	0.417
ΔPNS	Head	+0.042	0.169	+0.002	0.681
ΔSDNN	Neck	−0.998	0.049 *	+0.190	0.036 *
ΔRMSSD	Neck	−0.400	0.440	+0.160	0.092 †
ΔpNN50	Neck	−0.123	0.685	+0.083	0.094 †
ΔSNS	Neck	−0.017	0.657	+0.000	0.993
ΔPNS	Neck	+0.018	0.409	+0.001	0.749

Decomposition of strain into between-subject (subject-level mean; independent of experimental order) and within-subject (deviation from subject mean; collinear with sequence order) components entered simultaneously as predictors of ΔHRV in mixed-effects models. This decomposition separates effects attributable to anatomical variability from those potentially confounded by temporal adaptation or habituation. Significance: *p < 0.05; †p < 0.10.

**Table 5:** Changes in HRV-derived indices in the absence of cervical compression.

HRV index	n	Mean	SD	t-stat	p (t-test)	p (Wilcoxon)
ΔSDNN (ms)	17	−9.41	10.86	−3.57	0.003 *	0.004 *
ΔRMSSD (ms)	17	−10.68	12.63	−3.49	0.003 *	0.003 *
ΔpNN50 (%)	17	−5.57	7.36	−3.12	0.007 *	0.004 *
ΔSNS (a.u.)	17	+0.61	0.65	+3.85	0.001 *	0.001 *
ΔPNS (a.u.)	17	−0.30	0.43	−2.86	0.011 *	0.001 *

HRV changes from Baseline in the subset of observations with strain_neck ≤ 0 (8 Blue, 9 Yellow). Mean, standard deviation, one-sample t-statistic with associated p-value, and Wilcoxon signed-rank p-value against a null hypothesis of zero change are reported. Persistence of significant changes despite the absence of apparent neck compression suggests that direct cervical mechanical engagement alone is unlikely to explain the observed pattern. Significance: *p < 0.05.

## 4 Discussion

This pilot study explored whether wearing differently fitting wetsuit hoods under controlled dry conditions is associated with changes in selected HRV-derived indices. The principal finding was a reduction in RMSSD and pNN50 under hooded conditions, together with changes in Kubios-derived composite indices. However, interpretation of these findings as evidence of specific sympathetic or parasympathetic responses remains limited. Contemporary methodological literature emphasizes that HRV-derived measures should not be interpreted as direct indicators of autonomic branch activity or sympathovagal balance (Berntson et al., 1997; Eckberg, 1997; Goldberger, 1999; Houle and Billman, 1999; Malpas, 2002; Goldstein et al., 2011; Rahman et al., 2011; Billman, 2013a; Reyes del Paso et al., 2013; Heathers, 2014; Quintana et al., 2016; Laborde et al., 2017; Quigley et al., 2024). HRV reflects multiple interacting influences, including respiratory dynamics, baroreflex function, and non-reciprocal autonomic behavior (Berntson et al., 1997; Eckberg, 1997; Goldberger, 1999; Houle and Billman, 1999; Malpas, 2002; Goldstein et al., 2011; Rahman et al., 2011; Billman, 2013a; Reyes del Paso et al., 2013; Heathers, 2014; Quigley et al., 2024). Consequently, observed changes are more appropriately interpreted as alterations in cardiovascular variability rather than evidence of specific autonomic activation. The most consistent pattern involved reductions in RMSSD and pNN50, indices commonly associated with short-term vagally mediated HRV (Grossman and Taylor, 2007; Laborde et al., 2017). However, respiratory frequency was not measured, and respiratory influences on these findings cannot be excluded (Grossman and Taylor, 2007; Shattock and Tipton, 2012; Quintana and Heathers, 2014; Lundell et al., 2020; Lundell et al., 2021; Ackermann et al., 2023; Lundell and Ojanen, 2023; Quigley et al., 2024). Similarly, Kubios-derived SNS and PNS metrics should be interpreted as indirect descriptors of HRV patterns rather than direct measures of sympathetic or parasympathetic activity. The present findings indicate that wearing the Mephisto hood under dry conditions was associated with changes in selected HRV-derived indices. Because mechanical compression was not directly measured and experimental conditions followed a fixed sequence, these observations should be interpreted cautiously. Observed effects may reflect a combination of habituation, respiratory influences, discomfort, mechanical constraint, or other unmeasured factors. Exploratory analyses suggested an association between neck circumference and changes in the Kubios-derived SNS metric during the final hood exposure. However, neck circumference represents only an indirect proxy of fit, and continuous-strain analyses did not support a robust compression-specific dose–response relationship. Moreover, within-subject strain variation remained confounded by sequence order (Section 3.2; **Tables 3**–**5**). Taken together, these findings do not support attribution of observed HRV changes to specific physiological mechanisms. Interpretation of HRV-derived findings should additionally consider prevailing heart rate, as HR may mathematically influence HRV independently of physiological autonomic modulation (Billman, 2013b). Although Mean HR differed across conditions at the omnibus level, pairwise comparisons versus Baseline were not significant after correction, suggesting only modest variation. Future studies should therefore consider HR-corrected HRV measures or joint HR–HRV modelling approaches (Billman, 2013b).Future investigations incorporating randomized or counterbalanced designs, direct pressure measurements, respiratory monitoring, and complementary autonomic markers will be required to clarify the reproducibility and physiological basis of hood-associated HRV changes.

### 4.1 Compression–HRV relationship: interpretation of the continuous strain analysis

To complement categorical condition analyses in the absence of direct pressure measurements, we explored associations between HRV-derived changes and a continuous compression proxy based on geometric mismatch between subject anthropometry and hood dimensions. In pooled mixed-effects models, greater head strain was associated with smaller reductions in SDNN (β = +0.27, p = 0.041), with similar non-significant trends for RMSSD and pNN50. Because experimental conditions followed a fixed sequence (White → Blue → Yellow), within-subject strain variation was collinear with exposure order. Consequently, observed associations are compatible with both progressive habituation and alternative compression-related influences. Decomposition into between-subject and within-subject strain components did not provide robust evidence for compression-specific effects: only two of fourteen between-subject contrasts reached nominal significance, and statistical power for between-subject analyses was limited (N = 11). Furthermore, significant HRV-derived changes persisted in observations without apparent neck compression (strain_neck ≤ 0), suggesting that direct cervical mechanical engagement alone is unlikely to explain the observed pattern. Taken together, these findings do not support attribution of observed HRV changes to a specific physiological mechanism and do not permit discrimination between habituation, compression-related effects, or other unmeasured contributors. Future studies incorporating randomized or counterbalanced protocols together with direct pressure measurements will be necessary to clarify the reproducibility and physiological basis of hood-associated HRV changes.

## 5 Future perspectives

Future studies should incorporate randomized designs, direct pressure measurements, respiratory monitoring, and complementary physiological markers to clarify the reproducibility and physiological basis of hood-associated HRV changes. Standardized assessment of perceived discomfort may help distinguish mechanical from perceptual contributors. Future investigations may benefit from integrating complementary autonomic biomarkers beyond HRV, including pupillometric measures and analysis of pupillary oscillations, which have recently shown sensitivity to autonomic adjustments during face immersion and may improve multimodal characterization of physiological responses under diving-related conditions (Rizzuto et al., 2025a; Rizzuto et al., 2025b).

## 6 Limitations of the study

Several limitations should be considered when interpreting the present findings. First, the small sample size (N = 11), exclusive inclusion of male military personnel, and broad age range (30–61 years) limit generalizability and reduce power for between-subject analyses. Age-related effects on HRV were not modelled because statistical power was insufficient to support adjusted analyses. Second, experimental conditions followed a fixed sequence (Baseline → White → Blue → Yellow). Although washout periods and standardized timing were implemented, potential confounding by habituation, respiratory stabilization, and temporal adaptation cannot be excluded. Continuous-strain analyses further support this limitation, as strain variation was collinear with sequence order, preventing separation of within-subject compression effects from temporal adaptation. Third, mechanical compression was not measured directly. Compression was operationalized using geometric mismatch indices derived from participant anthropometry and hood dimensions. These proxies do not account for local pressure distribution, material stiffness, tissue compliance, or dynamic deformation and should therefore be interpreted as geometric surrogates rather than physical measurements. Direct pressure mapping and characterization of neoprene properties would improve physiological interpretation in future studies (Potočić Matković et al., 2025). Fourth, respiratory frequency was not measured, although respiratory sinus arrhythmia may influence RMSSD and related HRV indices. Fifth, frequency-domain measures (LF, HF, LF/HF, VLF) were not included among primary outcomes and were therefore not subjected to inferential analysis or logarithmic transformation. Alternative threshold-based HRV metrics (e.g., pNNx measures beyond pNN50) were also not explored, although such measures may provide complementary physiological information (Mietus et al., 2002). Sixth, recordings were limited to short-term dry laboratory conditions and do not reproduce environmental stressors associated with actual diving, including immersion, hydrostatic pressure, and thermal exposure. Consequently, extrapolation to operational diving settings should be made cautiously. Finally, interpretation of HRV findings should consider prevailing heart rate, as HR may mathematically influence HRV independently of physiological autonomic modulation (Billman, 2013b). Although Mean HR differed across conditions at the omnibus level, pairwise comparisons versus Baseline were not significant after correction. Future studies may therefore benefit from evaluating HR-corrected HRV measures or incorporating joint HR–HRV modelling approaches (Billman, 2013b).

## 7 Conclusions

In this pilot study, wearing wetsuit hoods under controlled dry laboratory conditions was associated with changes in selected HRV-derived indices compared with baseline recordings. The most consistent findings involved reductions in RMSSD and pNN50 across hooded conditions together with alterations in composite Kubios-derived metrics. In line with contemporary methodological recommendations, these findings should not be interpreted as direct evidence of specific sympathetic or parasympathetic activation (Berntson et al., 1997; Eckberg, 1997; Goldberger, 1999; Houle and Billman, 1999; Malpas, 2002; Goldstein et al., 2011; Rahman et al., 2011; Billman, 2013a; Reyes del Paso et al., 2013; Heathers, 2014; Quintana et al., 2016; Laborde et al., 2017; Quigley et al., 2024). Exploratory strain analyses did not support a robust compression-specific dose–response relationship. Accordingly, the observed HRV changes should be interpreted as exploratory and descriptive rather than mechanistic, as the present design does not permit attribution to specific physiological pathways and may reflect multiple unmeasured influences associated with hood use under controlled conditions. Future studies incorporating randomized or counterbalanced designs, direct pressure measurements, respiratory monitoring, and complementary physiological markers will be necessary to clarify the reproducibility and physiological basis of hood-associated HRV changes.

## Data Availability

The original contributions presented in the study are included in the article/**Supplementary Material**. Further inquiries can be directed to the corresponding author.
