# Supplementary material for: Heart rate variability changes associated with wetsuit hood fit in dry conditions: a pilot exploratory study
Source: Front Physiol. 2026 Jul 8;17:1759395. doi: 10.3389/fphys.2026.1759395 (PMC13388054; doi:10.3389/fphys.2026.1759395)
Supplement: Supplementary file 1 [file DataSheet1.docx]

**Supplementary Materials**

| **Parameter** | **Comparison** | **Baseline median [IQR]** | **White median [IQR]** | **Blue median [IQR]** | **Yellow median [IQR]** | **N_pairs** | **Wilcoxon_T** | **Z_value** | **p_unadjusted** | **p_Holm** | **r_effect** |
| --- | --- | --- | --- | --- | --- | --- | --- | --- | --- | --- | --- |
| **SDNN** | Baseline vs White | 40.40 [29.85–47.30] | 28.10 [22.30–40.10] |  |  | 11 | 20.0 | -1.1558 | 0.2783 | 0.5566 | -0.3485 |
| **SDNN** | Baseline vs Blue | 40.40 [29.85–47.30] |  | 27.10 [18.70–37.85] |  | 11 | 8.0 | -2.2228 | 0.0244 | 0.1221 | -0.6702 |
| **SDNN** | Baseline vs Yellow | 40.40 [29.85–47.30] |  |  | 27.20 [18.25–37.80] | 11 | 1.0 | -2.8451 | 2 | 0.0117 | -0.8578 |
| **SDNN** | White vs Blue |  | 28.10 [22.30–40.10] | 27.10 [18.70–37.85] |  | 11 | 17.0 | -1.4226 | 0.1748 | 0.5244 | -0.4289 |
| **SDNN** | White vs Yellow |  | 28.10 [22.30–40.10] |  | 27.20 [18.25–37.80] | 11 | 12.0 | -1.8671 | 0.0674 | 0.2695 | -563 |
| **SDNN** | Blue vs Yellow |  |  | 27.10 [18.70–37.85] | 27.20 [18.25–37.80] | 11 | 25.0 | -0.7113 | 0.5195 | 0.5566 | -0.2145 |
| **RMSSD** | Baseline vs White | 29.80 [25.95–35.10] | 21.00 [16.95–30.40] |  |  | 11 | 5.0 | -2.4895 | 0.0098 | 0.0488 | -0.7506 |
| **RMSSD** | Baseline vs Blue | 29.80 [25.95–35.10] |  | 22.70 [13.10–32.00] |  | 11 | 10.0 | -2.0449 | 42 | 168 | -0.6166 |
| **RMSSD** | Baseline vs Yellow | 29.80 [25.95–35.10] |  |  | 21.90 [12.45–29.35] | 11 | 2.0 | -2.7562 | 0.0029 | 0.0176 | -831 |
| **RMSSD** | White vs Blue |  | 21.00 [16.95–30.40] | 22.70 [13.10–32.00] |  | 11 | 27.0 | -0.5337 | 0.6201 | 0.6445 | -0.1609 |
| **RMSSD** | White vs Yellow |  | 21.00 [16.95–30.40] |  | 21.90 [12.45–29.35] | 11 | 16.0 | -1.5115 | 0.1475 | 0.4424 | -0.4557 |
| **RMSSD** | Blue vs Yellow |  |  | 22.70 [13.10–32.00] | 21.90 [12.45–29.35] | 10 | 17.0 | -1.0703 | 0.3223 | 0.6445 | -0.3384 |
| **PNN50** | Baseline vs White | 6.41 [4.37–13.63] | 3.40 [1.38–7.46] |  |  | 11 | 4.0 | -2.5784 | 0.0068 | 0.0342 | -0.7774 |
| **PNN50** | Baseline vs Blue | 6.41 [4.37–13.63] |  | 2.19 [0.41–9.62] |  | 11 | 11.0 | -1956 | 0.0537 | 0.1611 | -0.5898 |
| **PNN50** | Baseline vs Yellow | 6.41 [4.37–13.63] |  |  | 2.73 [0.09–7.05] | 10 | 0.0 | -2.8031 | 2 | 0.0117 | -0.8864 |
| **PNN50** | White vs Blue |  | 3.40 [1.38–7.46] | 2.19 [0.41–9.62] |  | 10 | 19.0 | -0.8664 | 0.4316 | 0.8633 | -274 |
| **PNN50** | White vs Yellow |  | 3.40 [1.38–7.46] |  | 2.73 [0.09–7.05] | 9 | 1.0 | -2.5471 | 0.0078 | 0.0342 | -849 |
| **PNN50** | Blue vs Yellow |  |  | 2.19 [0.41–9.62] | 2.73 [0.09–7.05] | 10 | 20.0 | -0.7645 | 0.4922 | 0.8633 | -0.2417 |
| **%PNS** | Baseline vs White | 28.30 [25.50–42.10] | 29.50 [26.15–32.45] |  |  | 11 | 20.0 | -1.1558 | 0.2783 | 1.0 | -0.3485 |
| **%PNS** | Baseline vs Blue | 28.30 [25.50–42.10] |  | 30.10 [26.45–32.35] |  | 9 | 22.0 | -0.0593 | 0.9844 | 1.0 | -0.0198 |
| **%PNS** | Baseline vs Yellow | 28.30 [25.50–42.10] |  |  | 28.90 [26.10–33.25] | 11 | 31.5 | -0.1334 | 915 | 1.0 | -0.0402 |
| **%PNS** | White vs Blue |  | 29.50 [26.15–32.45] | 30.10 [26.45–32.35] |  | 11 | 24.0 | -0.8002 | 0.4648 | 1.0 | -0.2413 |
| **%PNS** | White vs Yellow |  | 29.50 [26.15–32.45] |  | 28.90 [26.10–33.25] | 11 | 28.0 | -0.4446 | 0.7002 | 1.0 | -134 |
| **%PNS** | Blue vs Yellow |  |  | 30.10 [26.45–32.35] | 28.90 [26.10–33.25] | 10 | 26.0 | -0.1529 | 0.9219 | 1.0 | -0.0483 |
| **%SNS** | Baseline vs White | 71.70 [57.90–74.50] | 70.50 [67.55–73.85] |  |  | 11 | 20.0 | -1.1558 | 0.2783 | 1.0 | -0.3485 |
| **%SNS** | Baseline vs Blue | 71.70 [57.90–74.50] |  | 69.90 [67.65–73.55] |  | 9 | 22.0 | -0.0593 | 0.9844 | 1.0 | -0.0198 |
| **%SNS** | Baseline vs Yellow | 71.70 [57.90–74.50] |  |  | 71.10 [66.75–73.90] | 11 | 31.0 | -0.1778 | 0.8984 | 1.0 | -0.0536 |
| **%SNS** | White vs Blue |  | 70.50 [67.55–73.85] | 69.90 [67.65–73.55] |  | 11 | 24.0 | -0.8002 | 0.4648 | 1.0 | -0.2413 |
| **%SNS** | White vs Yellow |  | 70.50 [67.55–73.85] |  | 71.10 [66.75–73.90] | 11 | 28.0 | -0.4446 | 0.7002 | 1.0 | -134 |
| **%SNS** | Blue vs Yellow |  |  | 69.90 [67.65–73.55] | 71.10 [66.75–73.90] | 10 | 26.0 | -0.1529 | 0.9219 | 1.0 | -0.0483 |
| **PNS** | Baseline vs White | -0.64 [-0.95–0.23] | -0.65 [-1.25–0.26] |  |  | 11 | 15.0 | -1.6012 | 0.1172 | 0.5859 | -0.4828 |
| **PNS** | Baseline vs Blue | -0.64 [-0.95–0.23] |  | -0.65 [-0.99–0.28] |  | 11 | 22.0 | -0.9785 | 0.3535 | 1.0 | -295 |
| **PNS** | Baseline vs Yellow | -0.64 [-0.95–0.23] |  |  | -0.76 [-1.09–0.17] | 9 | 8.0 | -1.7193 | 0.0938 | 0.5625 | -0.5731 |
| **PNS** | White vs Blue |  | -0.65 [-1.25–0.26] | -0.65 [-0.99–0.28] |  | 9 | 14.0 | -1007 | 0.3594 | 1.0 | -0.3357 |
| **PNS** | White vs Yellow |  | -0.65 [-1.25–0.26] |  | -0.76 [-1.09–0.17] | 11 | 30.0 | -0.2669 | 0.8135 | 1.0 | -0.0805 |
| **PNS** | Blue vs Yellow |  |  | -0.65 [-0.99–0.28] | -0.76 [-1.09–0.17] | 11 | 26.0 | -0.6227 | 0.5625 | 1.0 | -0.1877 |
| **SNS** | Baseline vs White | 0.65 [-0.31–1.23] | 1.17 [-0.25–1.50] |  |  | 11 | 9.0 | -2.1349 | 0.0303 | 0.1514 | -0.6437 |
| **SNS** | Baseline vs Blue | 0.65 [-0.31–1.23] |  | 0.81 [-0.21–1.23] |  | 9 | 9.0 | -1.5993 | 0.1289 | 0.5156 | -0.5331 |
| **SNS** | Baseline vs Yellow | 0.65 [-0.31–1.23] |  |  | 1.08 [-0.35–1.81] | 11 | 5.0 | -2.4907 | 0.0098 | 0.0586 | -751 |
| **SNS** | White vs Blue |  | 1.17 [-0.25–1.50] | 0.81 [-0.21–1.23] |  | 11 | 18.0 | -1.3343 | 0.1973 | 0.5156 | -0.4023 |
| **SNS** | White vs Yellow |  | 1.17 [-0.25–1.50] |  | 1.08 [-0.35–1.81] | 11 | 26.0 | -0.6227 | 0.5605 | 0.5605 | -0.1877 |
| **SNS** | Blue vs Yellow |  |  | 0.81 [-0.21–1.23] | 1.08 [-0.35–1.81] | 11 | 15.5 | -1.5575 | 0.1289 | 0.5156 | -0.4696 |

**Supplemental Table 1:** Pairwise comparisons of HRV-derived indices across experimental conditions (Baseline, White, Blue, Yellow) using the Wilcoxon signed-rank test. For each comparison, median and interquartile range (IQR) are reported together with the number of valid pairs (N), Wilcoxon statistic (T), standardized Z value, unadjusted p-value, Holm-adjusted p-value, and effect size (r = Z/√N). Significant differences were observed for SDNN, RMSSD, pNN50, and SNS in selected comparisons. In contrast, %PNS and %SNS did not differ significantly across conditions. Trends approaching significance for some PNS comparisons should be interpreted cautiously given the exploratory nature of the analysis.

**Supplemental Table 2:** Individual anthropometric characteristics of enrolled participants (N = 11), including height, body weight, and calculated body mass index (BMI). These variables are reported to characterize the study population and provide context for potential interindividual variability in HRV-derived responses.

| **ID** | **Height_m** | **Weight_kg** | **BMI** |  |
| --- | --- | --- | --- | --- |
| 1 | 1.85 | 85 | 24.84 |  |
| 2 | 1.77 | 97 | 30.96 |  |
| 3 | 1.7 | 78 | 26.99 |  |
| 4 | 1.72 | 85 | 28.73 |  |
| 5 | 1.62 | 66 | 25.15 |  |
| 6 | 1.8 | 74 | 22.84 |  |
| 7 | 1.85 | 105 | 30.68 |  |
| 8 | 1.81 | 78 | 23.81 |  |
| 9 | 1.85 | 90 | 26.3 |  |
| 10 | 1.73 | 73 | 24.39 |  |
| 11 | 1.7 | 98 | 33.91 |  |

**Supplemental Table 3.** Mean heart rate (Mean HR), mean RR interval (Mean RR), minimum heart rate (HRmin), and maximum heart rate (HRmax) for each participant across Baseline, White, Blue, and Yellow conditions. Values are reported descriptively to contextualize interpretation of HRV-derived indices in light of the potential influence of prevailing heart rate on HRV measures. Mean HR differed across conditions at the omnibus level, although pairwise comparisons versus Baseline did not remain significant after correction for multiple testing.

| **PATIENT N.** | **SDNN_BASAL** | **SDNN_WHITE** | **SDNN_BLUE** | **SDNN_YELLOW** | **RMSSD _BASAL** | **RMSSD_WHITE** | **RMSSD_BLUE** | **RMSSD_YELLOW** | **PNN50_BASAL** | **PNN50_WHITE** | **PNN50_BLUE** | **PNN50_YELLOW** | **%PNS_BASAL** | **%PNS_WHITE** | **%PNS_BLUE** | **%PNS_YELLOW** | **%SNS_BASAL** | **%SNS_WHITE** | **%SNS_BLUE** | **%SNS_YELLOW** | **SNS_BASAL** | **SNS_WHITE** | **SNS_BLUE** | **SNS_YELLOW** | **PNS_BASAL** | **PNS_WHITE** | **PNS_BLUE** | **PNS_YELLOW** |
| --- | --- | --- | --- | --- | --- | --- | --- | --- | --- | --- | --- | --- | --- | --- | --- | --- | --- | --- | --- | --- | --- | --- | --- | --- | --- | --- | --- | --- |
| **1** | 19,6 | 20,4 | 15,7 | 15,2 | 13,2 | 13,1 | 10,6 | 9,6 | 0 | 0,21 | 0,22 | 0 | **26,4** | **25,3** | **26,4** | **25** | **73,6** | **74,7** | **73,6** | **75** | 1,23 | 1,27 | 1,23 | 1,96 | -0,64 | -0,65 | -0,65 | -0,76 |
| **2** | 40,5 | 46,3 | 42,1 | 35,4 | 29,6 | 31,5 | 33,4 | 28,1 | 9,04 | 8,17 | 13,72 | 7,14 | **28,3** | **26,6** | **30,2** | **30,2** | **71,7** | **73,4** | **69,8** | **69,8** | 0,8 | 0,51 | 0,53 | 1,08 | -1,03 | -0,86 | -0,78 | -1,03 |
| **3** | 54 | 72,2 | 73,1 | 54,6 | 59,1 | 62,5 | 67 | 60,7 | 37,45 | 38,61 | 39,89 | 35,45 | **39,7** | **32,5** | **34** | **41,1** | **60,3** | **67,5** | **66** | **58,9** | -1,01 | -0,69 | -0,81 | -1,17 | 1,18 | 0,67 | 0,89 | 1,11 |
| **4** | 27,4 | 28,1 | 23,2 | 20,7 | 37,2 | 29,3 | 30,6 | 30,1 | 15,57 | 6,28 | 5,  25 | 5,94 | **48** | **38** | **46,9** | **51,5** | **52** | **62** | **53,1** | **48,5** | -0,72 | -0,39 | -0,18 | -0,44 | 1,05 | 0,46 | 0,78 | 0,92 |
| **5** | 62,3 | 65,8 | 38,9 | 42,2 | 82,1 | 77,8 | 47,1 | 41,6 | 46,05 | 40,6 | 23,49 | 23,2 | **46,8** | **42,3** | **43,2** | **36,3** | **53,2** | **57,7** | **56,8** | **63,7** | -1,25 | -1,17 | -0,75 | -0,56 | 1,84 | 1,72 | 0,64 | 0,46 |
| **6** | 57,7 | 33,9 | 36,8 | 40,2 | 33 | 29 | 26,7 | 28,6 | 11,7 | 6,76 | 5,53 | 6,96 | **23** | **32,2** | **28** | **27,6** | **77** | **67,8** | **72** | **72,4** | 0,1 | -0,11 | -0,25 | -0,26 | -0,6 | 0,06 | -0,09 | -0,12 |
| **7** | 40,4 | 27 | 19,2 | 27,2 | 29,8 | 20,9 | 15,2 | 21,6 | 6,41 | 3,4 | 0,61 | 2,73 | **28,6** | **29,5** | **30,1** | **30,2** | **71,4** | **70,5** | **69,9** | **69,8** | 1,23 | 1,27 | 1,23 | 1,96 | -0,64 | -0,65 | -0,65 | -0,76 |
| **8** | 24,5 | 13,6 | 18,2 | 15,8 | 30,7 | 9,2 | 11 | 11,9 | 3,64 | 0 | 0,16 | 0 | **44,5** | **26,5** | **24,1** | **28,9** | **55,5** | **73,5** | **75,9** | **71,1** | 0,65 | 2,76 | 1,69 | 1,52 | -0,72 | -1,41 | -1,37 | -1,16 |
| **9** | 39,4 | 31,7 | 33,6 | 31,4 | 26,7 | 20,8 | 22,7 | 21,9 | 5,58 | 2,54 | 2,19 | 2,07 | **26,5** | **25,8** | **26,5** | **27,2** | **73,5** | **74,2** | **73,5** | **72,8** | 0,45 | 1,17 | 1 | 1,01 | -0,87 | -1,08 | -1 | -0,87 |
| **10** | 40,6 | 24,2 | 27,1 | 22,6 | 25,2 | 21 | 22 | 13 | 5,1 | 2,68 | 1,45 | 0,18 | **24,6** | **32,4** | **30,7** | **23,2** | **75,4** | **67,6** | **69,3** | **76,8** | 1,22 | 1,72 | 0,81 | 1,66 | -1,31 | -1,43 | -0,98 | -1,35 |
| **11** | 32,3 | 15,3 | 11,2 | 12,8 | 17,6 | 8,1 | 6,7 | 6,7 | 2,07 | 0 | 0 | 0 | **22** | **21,6** | **23,9** | **21,2** | **78** | **78,4** | **76,1** | **78,8** | 2,1 | 3,91 | 3,86 | 4,45 | -1,87 | -2,16 | -2,06 | -2,23 |

**Supplemental Table 4. Heart rate variability parameters (SDNN, RMSSD, pNN50, PNS, and SNS) measured across four experimental conditions (Baseline, White, Blue, and Yellow). The table illustrates variations in selected HRV-derived measures among conditions.**

**
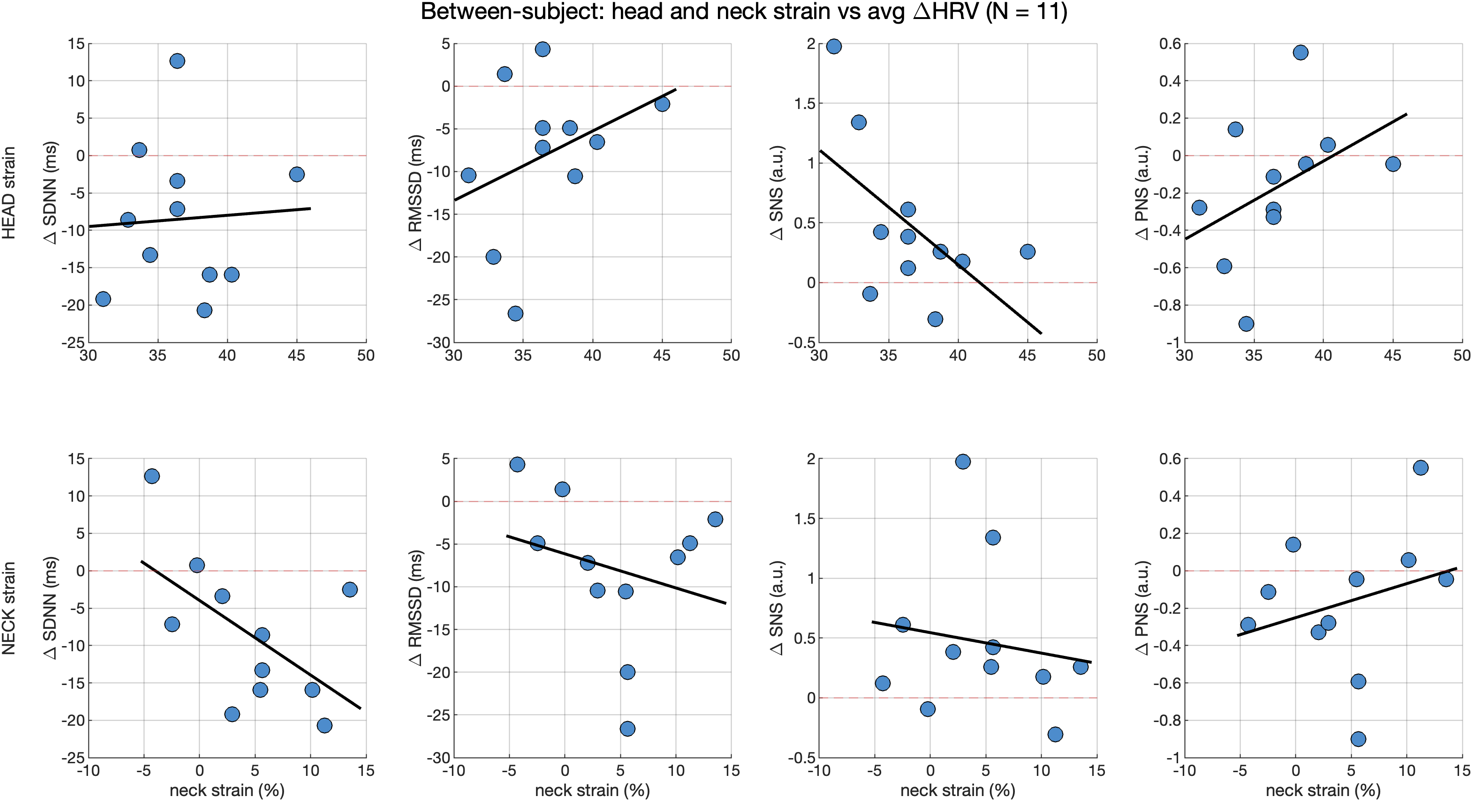
**

**Supplemental Figure 1.** Between-subject associations between head and neck strain and mean change-from-Baseline HRV-derived indices (N = 11). Each point represents one participant, with HRV responses averaged across hood conditions. Upper panels show associations with head strain; lower panels with neck strain. Solid black lines indicate least-squares fits and are shown for visualization purposes only; red dashed lines indicate no change from Baseline (Δ = 0). Given the exploratory design and limited sample size, these associations should be interpreted cautiously and do not imply causal relationships.
